# *MYOD1* (L122R) mutations are associated with spindle cell and sclerosing rhabdomyosarcomas with aggressive clinical outcomes

**DOI:** 10.1038/modpathol.2016.144

**Published:** 2016-08-26

**Authors:** Bharat Rekhi, Pawan Upadhyay, Manoj P Ramteke, Amit Dutt

**Affiliations:** 1 Department of Surgical Pathology, Tata Memorial Centre, Parel, Mumbai, Maharashtra, India; 2 Integrated Genomics Laboratory, Advanced Centre for Treatment, Research and Education In Cancer, Tata Memorial Centre, Navi Mumbai, Maharashtra, India; 3 Homi Bhabha National Institute, Training School Complex Anushakti Nagar, Mumbai, India

## Abstract

Recurrent mutations in the myogenic transcription factor *MYOD1* and *PIK3CA* were initially described in a subset of embryonal rhabdomyosarcomas. Recently, two independent studies demonstrated presence of *MYODI* (L122R) mutations as the basis to re-classify a spindle cell rhabdomyosarcoma, along with a sclerosing rhabdomyosarcoma, distinct from an embryonal rhabdomyosarcoma. We analyzed a much larger cohort of 49 primary rhabdomyosarcoma tumor samples of various subtypes, collected over a period of 9 years, for the presence of *MYOD1* (L122R), *PIK3CA* (H1047), and *PIK3CA* (E542/E545) mutations, along with immunohistochemical analysis of desmin, myogenin, and MYOD1. Although activating *PIK3CA* mutations were absent across the sample set analyzed, we report 20% *MYOD1* (L122R) mutation in rhabdomyosarcomas, found exclusively in 10 of 21 spindle cell and sclerosing rhabdomyosarcomas, occurring mostly in the head and neck region along with extremity sites (64%), than the paratesticular and intra-abdominal sites. Furthermore, while all 10 *MYOD1* mutant spindle cell and sclerosing rhabdomyosarcoma samples showed diffuse and strong MYOD1 immunoexpression, 7 of 31 samples of rhabdomyosarcoma with wild-type *MYOD1* were negative for MYOD1 expression. Clinically, a striking correlation was found between *MYOD1* mutation and the clinical outcomes available for 15 of 21 cases: 5 of 7 patients with spindle cell and sclerosing rhabdomyosarcomas, harboring *MYOD1* mutation, were alive-with-disease and 2 of 8 patients with spindle cell and sclerosing rhabdomyosarcomas, with mutant *MYOD1,* were free-of-disease. Taken together, we present the first report of *MYOD1* (L122R) mutation in the largest cohort of 49 rhabdomyosarcomas reported so far, that are associated with a relatively aggressive clinical course. Moreover, consistent with the earlier two studies, this study further reinforces a relationship between spindle cell and the sclerosing rhabdomyosarcoma—now recognized as a single subtype, distinct from an embryonal rhabdomyosarcoma.

Rhabdomyosarcoma (RMS), a rare malignant tumor of skeletal muscle origin, is presently subtyped into an embryonal RMS, an alveolar RMS; a pleomorphic RMS and more recently, spindle cell and sclerosing RMS.^1^ Spindle cell RMS, initially considered as a variant of an embryonal RMS, is commonly identified in the paratesticular region of pediatric patients and is associated with a relatively better clinical outcome.^2, 3^ Subsequently, cases of spindle cell RMS were also described in adult patients, associated with a relatively more aggressive clinical course.^4, 5, 6^ Sclerosing RMS was identified as another distinctive variant of a RMS, histopathologically mimicking an extraskeletal osteosarcoma, an extraskeletal myxoid chondrosarcoma, a sclerosing epithelioid fibrosarcoma, and an angiosarcoma.^7, 8, 9^ Several studies have described a relationship between spindle cell and sclerosing RMSs that lately have emerged together as a distinct subtype of a RMS.^4, 5, 10, 11, 12^ Certain studies also demonstrated a relationship between some cases of embryonal RMS and spindle cell and sclerosing RMS.^10, 12, 13^

Various investigators have unraveled genetic events underlying cases of spindle cell and sclerosing RMS. Kohsaka *et al*^14^ initially described a recurrent *MYOD1* (L122R) mutation defining a clinical subset of embryonal RMSs associated with *PI3K-AKT* pathway mutations. More recently, two independent studies demonstrated recurrent *MYOD1* in adult spindle cell RMSs,^15^ and pediatric and adult sclerosing RMSs^16^ reinforcing a relationship between these two morphological variants of a RMS.

Here, we present a systematic analysis of activating mutations in *MYOD1* and *PIK3CA* along with the expression of desmin, MYOD1, and myogenin in a much larger cohort of 49 primary samples of RMS collected over a period of 9 years: comprising of 21 spindle cell and sclerosing RMSs, 10 embryonal RMS,s 17 alveolar RMSs, and a single case of a pleomorphic RMS.

## Materials and methods

### Samples and Patients Description

The 49 cases of RMS included in the present study were selected, based on the availability of formalin-fixed and paraffin-embedded blocks, subsequent to a critical histopathologic review of 300 consecutive cases of RMS, diagnosed over a period of 9 years (2005–2013) at Tata Memorial Centre, Mumbai. Diagnostic criteria for various subtypes of RMS, including spindle cell/sclerosing RMS, were according to the recent World Health Organization classification of soft tissue tumors.^1^ Few cases of spindle cell RMS displayed focal areas of sclerosing RMS and *vice versa*. In such cases, the tumor was designated as spindle cell or sclerosing RMS, depending upon predominant tumor pattern present. All cases were reviewed by Bharat Rekhi, a pathologist, and the cases with an adequate amount of tumor content (more than 70%) in the corresponding paraffin blocks were included. Clinical and follow-up details were obtained with the help of electronic medical records, as well as telephonically.

### Immunohistochemistry

Immunohistochemical staining was performed on formalin-fixed paraffin-embedded tissue sections by immunoperoxidase method using a MACH 2 Universal HRP-Polymer detection kit (Biocare, CA, USA), including 3′-3′-diaminobenzidine tetrahydrochloride as the chromogen. Appropriate positive and negative controls were included. The details of various antibody markers, including dilution and manufacturers, are enlisted in Supplementary Table 3. Immunohistochemical studies were performed on all cases.

### Genomic DNA Extraction, PCR, and Sanger Sequencing

Genomic DNA from formalin-fixed and paraffin-embedded tissue blocks was isolated using QIAamp DNA formalin-fixed and paraffin-embedded tissue kit (Qiagen), as per manufacturer's instructions. DNA concentration was determined by absorbance at 280 nm (NanoDrop 2000c, Thermo Scientific). Primers used for PCR amplifications of *MYOD1* and *PIK3CA* amplicons are enlisted in Supplementary Table 4. PCR was performed in 25 *μ*l reaction volume containing 50–100 ng of genomic DNA from formalin-fixed and paraffin-embedded blocks with 0.2 *μ*M each of primer pairs using Veriti 96-Well Thermal Cycler (Applied Biosystems). For *MYOD1* L122 and *PIK3CA* H1047 amplicons, PCR was carried out with initial hot-start denaturation at 95 °C for 5 min, followed by 35 cycles of denaturation at 95 °C for 30 s, annealing at 55 °C for 30 s, polymerization at 72 °C for 45 s, and final incubation for 10 min at 72 °C. For *PIK3CA* E542/E545 amplicon, PCR was carried out using all conditions as mentioned above with the annealing temperature of 60 °C for 30 s. PCR amplicons were purified using Nucleospin gel and PCR clean-up kit (Macherey-Nagel). Sequencing of purified PCR products was performed by Sanger sequencing and data were analyzed using Mutation Surveyor software V4.0.9.^17^

### Statistical Analysis

Statistical analysis for various clinical features was carried out using IBM SPSS statistics software version 21 and significant differences were calculated using *χ*^2^ and Fisher's exact test. Threshold for statistical significance was set at *P*≤0.05.

## Results

**Figure 1:**
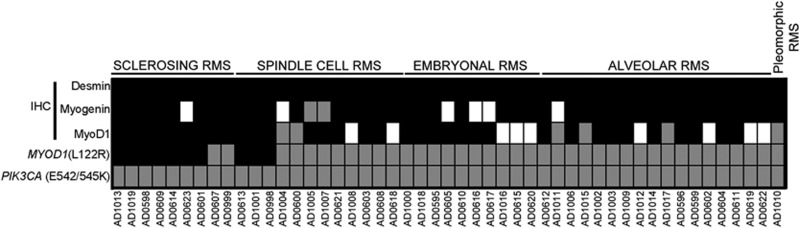
Immunohistochemical and mutational analysis of 49 rhabdomyosarcoma (RMS) cases. Schematic representation of subtypes of RMS and overview of immunohistochemistry of desmin, myogenin and MyoD1 and *MYOD1* (L122R), and *PIK3CA* (E542/E545 and H1047) mutation analysis. Black fill denotes sample harboring *MYOD1* mutation; gray denotes wild-type *MYOD1*; and white denotes for data not available.

Forty-nine cases of RMS in the present study included 17 cases of alveolar RMS, 10 of embryonal RMS, 21 of spindle cell and sclerosing RMS, and a single case of a pleomorphic RMS (Figure 1). Three out of 10 cases of alveolar RMS were tested for *PAX-FOXO1* fusion transcript by RT-PCR and were found to be positive for *PAX3-FOXO1* in two cases and *PAX7-FOXO1* in a single case. Clinicopathologic features of 21 cases of spindle cell and sclerosing RMSs are enlisted in Table 1. There were 18 males and 3 females (6:1). Age range was 2–66 years. Mean age was 19.6 years and median age was 19 years. Site-wise, the tumors occurred in the head and neck region,^11^ paratesticular region,^4^ extremities,^2^ intra-abdominal region,^3^ and in the chest wall.^1^ Twenty-one cases of spindle cell and sclerosing RMS comprised 12 cases of spindle cell and 9 cases of sclerosing RMS.

### Histopathologic and Immunohistochemical Characteristics of the Primary Rhabdomyosarcomas

**Figure 2:**
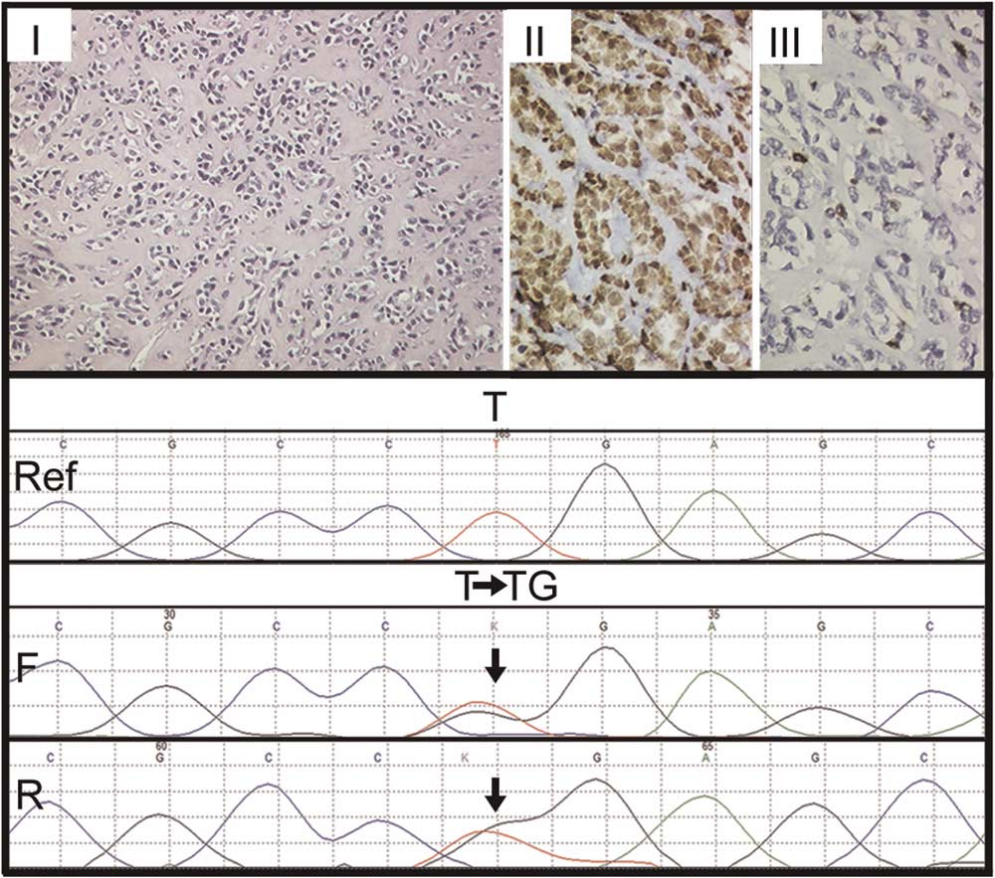
Immunohistochemical and mutational analysis of sclerosing rhabdomyosarcomas. Representative images of immunohistochemical analysis of MYOD1, myogenin, and *MYOD1* (L122R) mutation in sclerosing rhabdomyosarcomas. Upper panel: Case 1. Sclerosing rhabdomyosarcoma (RMS) (I–III). Round to oval cells in a pseudovascular- and cord-like patterns in a dense hyalinized stroma. Hematoxylin and Eosin (H and E), x200. II. Diffuse MYOD1-positive immunostaining. Diaminobenzidine, x400. III. Focal myogenin-positive immunostaining. Diaminobenzidine, x400. Lower panel: Sanger sequencing chromatogram of *MYOD1* (L122R, T>G or T>TG) is shown with reference sequences and mutation showing forward and reverse sequencing reads. Arrow indicates nucleotide position that harbors mutation.

**Figure 3:**
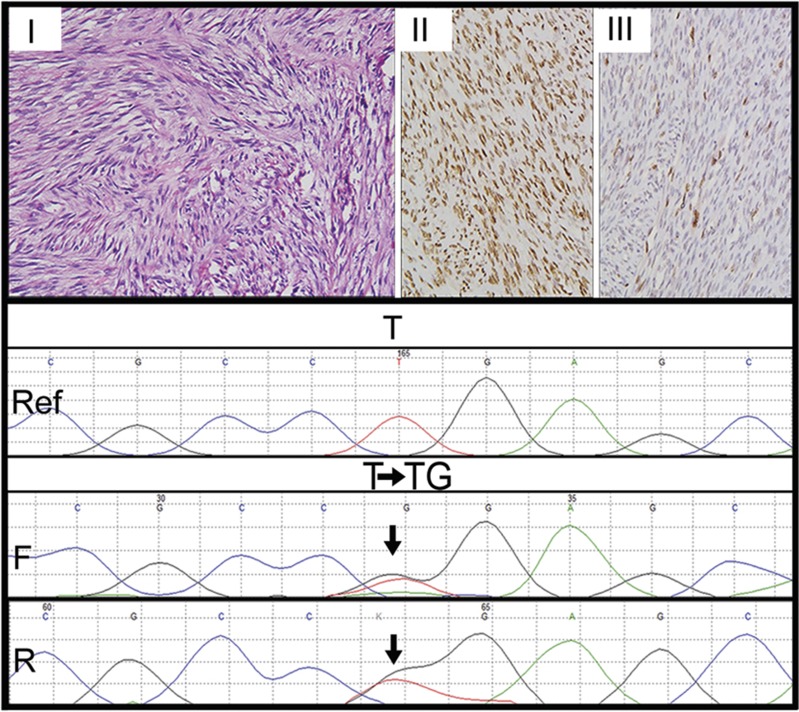
Immunohistochemical and mutational analysis of spindle cell rhabdomyosarcomas. Representative images of immunohistochemical analysis of MYOD1, myogenin, and *MYOD1* (L122R) mutation in spindle cell rhabdomyosarcomas. Upper panel: Case10. Spindle cell rhabdomyosarcoma (I–III). I. Spindle-shaped sarcomatous cells arranged in long intersecting fascicles. Hematoxylin and Eosin (H and E) x200. II. Diffuse MYOD1-positive immunostaining. Diaminobenzidine, x400. III. Myogenin-positive immunostaining. Diaminobenzidine, x400. Lower panel: Sanger sequencing chromatogram of *MYOD1* (L122R, T>G or T>TG) is shown with reference sequences and mutation showing forward and reverse sequencing reads. Arrow indicates nucleotide position that harbors mutation.

**Figure 4:**
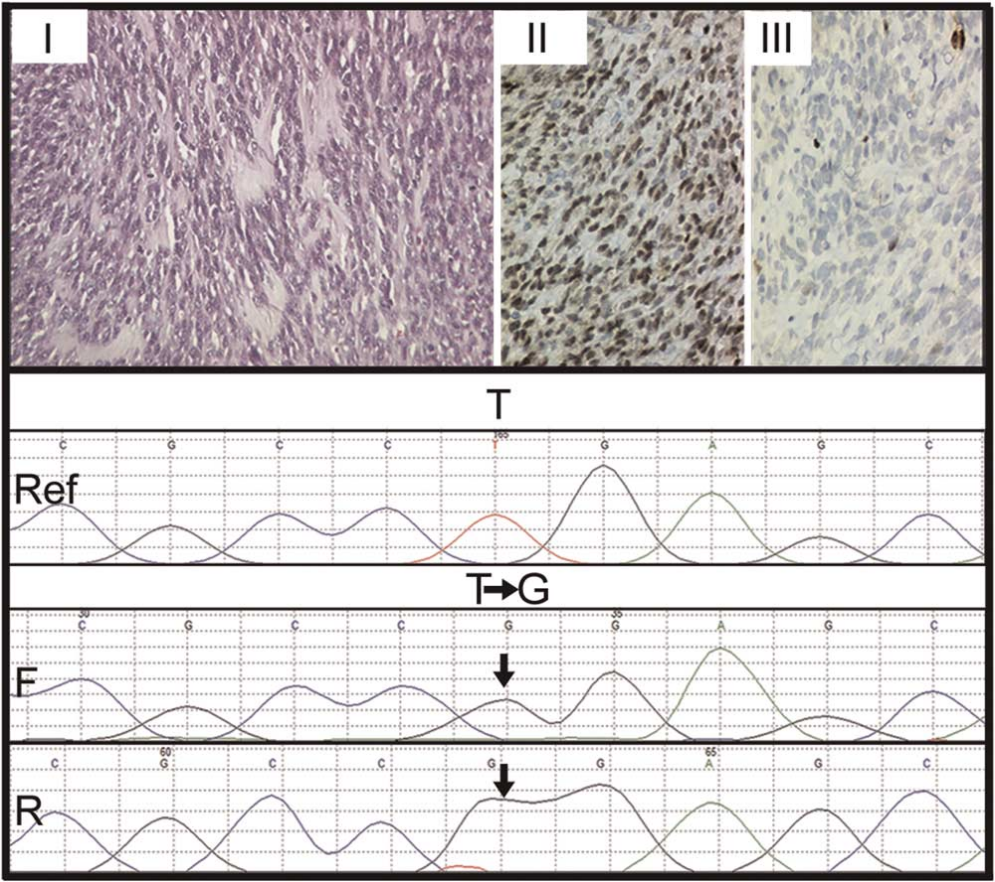
Immunohistochemical and mutational analysis of a sclerosing rhabdomyosarcoma with focal areas of spindle cell rhabdomyosarcoma. Representative images of immunohistochemical analysis of MYOD1, myogenin, and *MYOD1* (L122R) mutation in sclerosing rhabdomyosarcoma with focal spindle cell rhabdomyosarcomas. Case 2. Sclerosing rhabdomyosarcoma with focal spindle cells (I–III). I. Oval- to spindle-shaped cells in a dense hyalinized stroma. Hematoxylin and Eosin (H and E) x400. II. Diffuse MYOD1-positive immunostaining. Diaminobenzidine, x400. III. Focal myogenin-positive immunostaining. Diaminobenzidine, x400. Lower panel: Sanger sequencing chromatogram of *MYOD1* (L122R, T>G or T>TG) is shown with reference sequences and mutation showing forward and reverse sequencing reads. Arrow indicates nucleotide position that harbors mutation.

Histopathologically, cases of sclerosing RMS revealed pseudovascular-, nesting-, focal alveolar-, and cord-like arrangement of round to oval to short spindle-shaped tumor cells in a prominent hyaline or pseudochondroid stroma. Despite pseudoalveolar pattern noted in some cases of sclerosing RMS, none of those tumors displayed 'wreath-like' giant cells (Figure 2). Cases of spindle cell RMS displayed tumor cells with spindle-shaped nuclei, mostly arranged in long intersecting fascicles and occasionally in whorling patterns (Figure 3). Four cases of sclerosing RMS also displayed focal areas of tumor with spindle-shaped tumor cells (Figure 4). Certain cases of spindle cell and sclerosing RMS also displayed features of embryonal RMS, in the form of rhabdomyoblasts, especially cases of spindle cell RMS in tumors occurring in the paratesticular region. By immunohistochemistry, tumor cells in 21 of 21 cases were positive for desmin; in 18 of 20 were positive for MYOD1 in a diffuse pattern; in 17 of 19 were positive for myogenin (Figure 1); and in 6 of 11 cases were positive for smooth muscle actin, mostly in a focal pattern. In all cases, the tumor cells were positive for at least a single skeletal muscle-specific marker, namely MYOD1 and or myogenin. In a remaining single case, wherein MYOD1 was negative and myogenin was not performed, tumor cells were positive for myoglobin.

### Molecular Profiling for *MYOD1* and *PIK3CA* Mutations across 49 Rhabdomyosarcomas

*MYOD1* (L122R) mutation was found in 10 of 49 cases of RMS (Figure 1). Age-wise, 8 out of 10 cases were adult patients (>18 years of age) and 2 were pediatric patients (≤18 years of age) (*P*=0.08). Site-wise, 7 out of 10 cases revealing *MYOD1* (L122R) mutation occurred in the head and neck region, whereas 2 cases occurred in the extremities and a single case occurred in the chest wall. (Table 1). Out of these 10 cases displaying *MYOD1* mutation, 8 showed heterozygous mutations and 2 cases showed homozygous mutations. (Supplementary Figure 1 and Supplementary Table 1). All 10 cases displaying *MYOD1* (L122R) mutation were of spindle cell and sclerosing RMS. None of the remaining 10 cases of embryonal RMS and 17 cases of alveolar RMS and the single case of pleomorphic RMS displayed *MYOD1* (L122R) mutation. None of the 49 cases displayed *PIK3CA* (E542/E545) and *PIK3CA* (H1047) mutations (Figure 1). Among 21 cases of spindle cell and sclerosing RMS, more cases of sclerosing RMS (7/9) (78%) revealed *MYOD1* mutations, as compared with spindle cell RMS (3/12) (25%) (*P*=0.03). Among seven cases of sclerosing RMS displaying *MYOD1* mutations, four cases also displayed focal areas of tumor with spindle-shaped tumor cells. The remaining 11 cases of spindle cell and sclerosing RMS that showed wild-type *MYOD1* included 9 cases of spindle cell RMS and 2 cases of sclerosing RMS. Seven of these 9 cases of spindle cell RMS occurred in abdominal sites, including 3 cases in the retroperitoneum and mesentery and 4 in the paratesticular region. The remaining two cases occurred in the head and neck region including face and soft palate. The two cases of sclerosing RMS, revealing wild-type *MYOD1* occurred in the head and neck region (parotid and orbit) (Table 1).

### Clinical Outcomes and their Correlation with Age and *MYOD1* Mutational Status in Spindle Cell and Sclerosing Rhabdomyosarcoma

Treatment details were available in 19/21(90%) cases of spindle cell and sclerosing RMS. All the patients underwent surgical resections with adjuvant chemotherapy and radiotherapy in 9 cases; adjuvant chemotherapy (7 cases) and adjuvant radiotherapy (3 cases). Follow-up details (more than or equal to 6 months) were available in 15/21 cases (71%), over a period of 6–50 months (average=22 months, median=23 months). Overall, seven patients were alive-with-disease (over a duration of 6–33 months) and eight patients were free-of-disease (6–50 months) during the last follow up at our hospital. On comparing clinical outcomes between pediatric and adult patients, more number of adult patients was alive with disease (6/7) (86%), as compared with pediatric patients (1/7) (14%). Of the seven patients alive with disease, five were positive for *MYOD1* mutation (71%), whereas two patients lacked *MYOD1* mutation (29%). On the other hand, of eight patients free of disease, six were positive for wild-type *MYOD1* (75%), whereas two patients were positive for *MYOD1* mutation (25%). However, no statistically significant correlation (*P*=0.13) was observed between alive with disease and free of disease patients, with regard to *MYOD1* mutation status (Table 1). Of these two cases, one presented with pulmonary metastasis, and other with pulmonary and lymph node metastasis (Supplementary Tables 1 and 2).

## Discussion

The present study describes clinicopathologic features and analysis of *MYOD1* (L122R) and *PIK3CA* (E542/E545, H1047) mutation profiling in 21 cases of spindle cell and sclerosing RMS, including 12 adult and 9 pediatric patients. These mutations were also tested in 17 cases of alveolar RMS; 10 cases of embryonal RMS; and in a single case of pleomorphic RMS.

Histopathologically, there were 12 cases of spindle cell RMS and 9 of sclerosing RMS. Four cases of sclerosing RMS also displayed focal areas of tumor with spindle cells, as noted earlier.^8, 10, 11, 12^ Whereas fascicular pattern was the most commonly observed tumor pattern in cases of spindle cell RMS, pseudovascular pattern was most commonly observed in cases of sclerosing RMS.^4, 5, 7, 8, 9, 10, 11^ By immunohistochemistry, all 21 cases displayed desmin positivity, along with MyoD1 positivity in 90% cases and myogenin positivity in 90% cases. Noteworthy, MyoD1 immunoexpression was diffuse in most cases of sclerosing RMS, as previously observed.^10^

While none of the cases of embryonal RMS, alveolar RMS, or pleomorphic RMS revealed *MYOD1* mutation, consistent with the earlier reports;^18^ 10 of 21 (48%) cases of spindle cell and sclerosing RMS were found to harbor *MYOD1* (L122R) mutation, consistent with the earlier two reports wherein Szuhai *et al*^15^ reported the same in 41% cases of spindle cell RMS and Agaram *et al*^16^ reported this mutation in 56% cases of spindle cell and sclerosing RMS. Earlier, Kohsaka *et* *al*^14^ demonstrated *MYOD1* (L122R) mutation in 10% cases of embryonal RMS.

Unlike the results from a study by Szuhai *et al*,^15^ wherein all seven the cases displayed homozygous mutation of *MYOD1* (L122R), our study, similar to that of Agaram *et al*^16^ revealed cases of spindle cell and sclerosing RMS with heterozygous, as well as homozygous *MYOD1* L122R mutations. Furthermore, similar to the latter study, we observed *MYOD1* (L122R) mutation significantly more in the cases of sclerosing than spindle cell RMS (*P*=0.03).^16^ However, unlike Agaram *et al*,^16^ who observed *MYOD1* mutation in all five cases of sclerosing RMS and in 36% cases of spindle cell RMS, we observed the same in 78% cases of sclerosing RMS and in 25% cases of spindle cell RMS. We believe the possible reason for the lower mutation frequency in cases of spindle cell RMS in the present study might be because there were four cases of paratesticular and three cases of abdominal spindle cell RMS, all that are generally not associated with *MYOD1* (L122R) mutation, as recently described.^19^ A higher rate of *MYOD1* mutation in cases of spindle cell RMS, reported in an earlier study, is attributable toward the fact most of those tumors from extremity sites, rather than intra-abdominal and paratesticular sites.^15^ Thus, the present study showed association of *MYOD1* mutation, more with cases of spindle cell and sclerosing RMSs occurring in the head and neck and extremity sites, in contrast to those, especially with spindle cell morphology, occurring in the paratesticular and intra-abdominal sites, as noted in the two recent studies.^16, 19^

Therapeutically, most cases in the present study were treated with surgery and adjuvant chemotherapy with/without radiotherapy. On comparing clinical outcomes between adult and pediatric patients, more number of cases alive-with-disease was identified in the former group versus the latter. Though, not statistically significant, this suggests a trend for the spindle cell and sclerosing RMSs in adult patients to be relatively more clinically aggressive (*P*=0.08). This was likely as a result of 4/9 pediatric tumors in the present study occurring in paratesticular and intra-abdominal locations, wherein these tumors are associated with relatively better clinical outcomes.^19^ Furthermore, on comparing clinical outcomes in the cases showing *MYOD1* mutation versus those lacking this mutation, we observed that a higher number of cases (5/7) (71%) with disease during their last follow up, in the form of recurrences and or metastasis were observed in *MYOD1* mutant cases, although the difference was statistically not significant (*P*=0.34). Among the cases free-of-disease, there were less number of *MYOD1* mutant cases (2/8) (25%) than those lacking this mutation (6/8) (75%).

On comparing clinical outcomes between *MYOD1* mutant adult versus pediatric cases, more cases (5/6) (83%) alive-with-disease were seen in adult than in pediatric patients (1/6) (17%). Furthermore, more number of *MYOD1* mutant cases, alive-with-disease were seen in adult than pediatric patients, indicating a relatively more aggressive clinical course in the former than the latter group. Contrastingly, Agaram *et al*^16^ observed a relatively more aggressive clinical course of *MYOD1* mutant cases of spindle cell and sclerosing RMS in pediatric than in adult cases.^16^ This was in view of all such cases in their study group occurring sites other than paratesticular or abdominal region. Recently, Alaggio *et al*^19^ identified novel and recurrent *VGLL2*-related fusions in certain cases of infantile spindle cell RMS. Previously, Mosquera *et al*^20^ demonstrated recurrent *NCOA2* gene rearrangement in cases of congenital/infantile spindle cell RMS. The same authors observed a relationship between *NCOA2*-rearranged spindle cell RMS occurring in young childhood and the so-called congenital RMS, which are associated with rearrangements at 8q13 locus (*NCOA2*), suggesting spindle cell RMSs as a heterogeneous group of tumors.

Furthermore, a small subset of cases of sclerosing RMS is known to be associated with *PIK3CA* mutations. Shukla *et al*^21^ reported the presence of *PIK3CA* mutation in 5% cases (3/60) of embryonal RMS, two of which were re-classified as sclerosing RMS. Although varying frequency of activating somatic mutations across different populations is known in the literature for *EGFR*,^22^ *KRAS*,^23^ *BRAF*,^24^ and *PIK3CA*^25, 26^ in lung, colorectal, and other cancers, absence of *PIK3CA* mutations in Indian cases of RMS is surprising. Of note, given low sample size of 49 tumors, the current study might not be adequately statistically powered to detect its occurrence if it exists at an altered frequency lower than 2–3%. However, to explore the therapeutic implication of PI3 kinase inhibitors in cases of RMS of Indian ethnicity, alterations in *PTEN* and *AKT* affecting alternate or redundant mechanism of PI3 kinase pathway activation need to be further analyzed.^27, 28^

In summary, the presence of specific *MYOD1* (L122R) mutation in significant number of cases of spindle cell and sclerosing RMS, as noted in the present study, further reinforces a relationship between these two histopathological subtypes of RMS, now recognized as a single subtype of a RMS. Absence of this mutation in cases of embryonal RMS, RMS, and pleomorphic RMS indicates lack of the genetic relationship between spindle cell and sclerosing RMS with embryonal RMS that was previously considered, despite overlapping histopathologic and immunohistochemical features in some cases. MYOD1 immunostaining is frequently diffuse in cases of sclerosing RMS and constitutes as a useful immunohistochemical marker in these cases. *MYOD1* (L122R) mutation was found to be more frequent in case of sclerosing RMS (irrespective of pediatric or adult patients), than in the spindle cell RMS, that constitutes as a relatively more heterogeneous group. Cases of spindle cell RMS occurring in the head and neck and extremity locations, in adult patients have higher frequency of *MYOD1* mutation that those occurring in the paratesticular or intra-abdominal sites. *PIK3CA* mutations are rare in cases of spindle cell and sclerosing RMS and were not identified in any of our cases. Identification of specific *MYOD1* (L122R) mutations in cases of sclerosing/spindle cell RMS probably indicates a more intensive treatment, as these cases are associated with a relatively aggressive clinical course. Thus genotyping for *MYOD1* (L122R) mutation may preclude the requirement to perform immunohistochemical analysis in order to identify an aggressive subset of spindle cell and sclerosing RMSs, early on to help inform adoption of appropriate therapeutic regimen. At the same time, this discovery also seems to provide a direction toward avenues for targeted therapy in these cases.

## Figures and Tables

**Table 1:** Correlation between clinicopathologic features of spindle cell and sclerosing RMS and *MYOD1* mutation

*Clinicopathologic features*	*Variable*	*Frequency* (N*=21*)	*MYOD1 (L122R)*		χ*^2^-value*	P*- value*
		***19.6* (2–66)**	*Wild*	*Mutant*		
Age	>18 (adult)	12 (57%)	4 (12)	8(12)	4.07	**0.08**
	≤18 (pediatric)	9 (43%)	7 (9)	2(9)		
Sex	Male	18 (86%)	9 (18)	9 (18)	0.28	1
	Female	3(14%)	2 (3)	1 (3)		
Sites	Head and neck	11 (52%)	4 (11)	7 (11)	2.37	0.19
	Other sites	10 (48%)	7 (10)	3 (10)		
Histopathologic subtype	Spindle cell	12 (57%)	9 (12)	3 (12)	5.74	**0.03**
	Sclerosing	9 (43%)	2 (9)	7 (9)		
MYOD1 immunohistochemistry	Positive	17 (80%)	7 (17)	10 (17)	4.49	**0.09**
	Negative	2 (10%)	4 (4)	0 (4)		
	Data not available	2 (10%)				
Recurrence/Mets	Yes	10 (47%)	3 (10)	7 (10)	1.9	0.34
	No	8 (38%)	5 (8)	3 (8)		
	Data not available	3 (14%)				
Outcome	Alive with disease	7 (33%)	2 (7)	5 (7)	3.23	0.13
	Free of disease	8 (38%)	6 (8)	2 (8)		
	Data not available	4 (19%)				
	Recent (FU< 6 months)	2 (10%)				

Abbreviation: FU: follow up.

Clinicopathologic features, including results of *MYOD1* (L122R) mutations in 21 cases of spindle cell and sclerosing rhabdomyosarcomas. Highlighted *P*-values are statistically significant or of marginal significance.
