# Supplementary material for: MYOD1 (L122R) mutations are associated with spindle cell and sclerosing rhabdomyosarcomas with aggressive clinical outcomes
Source: Mod Pathol. 2016 Aug 26;29(12):1532–40. doi: 10.1038/modpathol.2016.144 (PMC5133269; doi:10.1038/modpathol.2016.144)
Supplement: Supplementary Information [file modpathol2016144x1.doc]

**Supplementary File Legends:**

**Supplementary Figure 1. *MYOD1* (L122R) mutation positive cases of** rhabdomyosarcoma **with their corresponding immunohistochemical results, including MYOD1 and Myogenin immunostaining**.

**Upper panel**

**A.** Case 3(I-III). I. Sclerosing rhabdomyosarcoma, including round to oval cells in a pseudochondroid stroma. H and E, x 200. II. Diffuse MYOD1 positive immunostaining. Diaminobenzidine, x400. III. Focally positive myogenin immunostaining. Diaminobenzidine, x 400.

**B.** Case 4(I-III). I. Sclerosing rhabdomyosarcoma (post chemotherapy). H and E, x 200. II. Diffuse MYOD1 positive immunostaining. Diaminobenzidine, x400. III. Focal positive Myogenin immunostaining. Diaminobenzidine, x 400.

**C.** Case 5(I-II). I. Sclerosing rhabdomyosarcoma with focal spindle cells. II. Higher magnification showing round to oval to spindle shaped cells in a hyalinized stroma.

**D.** Case 6 (I-III). I. Sclerosing rhabdomyosarcoma, including round to oval cells in a hyalinized stroma. H and E, x 200. II. Diffuse MYOD1 positive immunostaining. Diaminobenzidine, x400. III. Focal positive Myogenin immunostaining. Diaminobenzidine, x 400.

**E.** Case 7 (I-III). I. Sclerosing rhabdomyosarcoma. H and E, x 200. II. Diffuse MYOD1 positive immunostaining. Diaminobenzidine, x400. III. Focal positive Myogenin immunostaining. Diaminobenzidine, x 400.

**F**. (I-III). Case 8. (I-III). I. Spindle cell rhabdomyosarcoma, including elongate rhabdomyoblasts. H and E, x 200. II. Diffuse MYOD1 positive immunostaining. Diaminobenzidine, x400. III. Positive Myogenin positive immunostaining. Diaminobenzidine, x 400.

**G.** Case 9. (I-III). I. Spindle cell rhabdomyosarcoma. H and E, x 200. II. Diffuse MYOD1 positive immunostaining. Diaminobenzidine, x400. III. Diffuse myogenin positive immunostaining. Diaminobenzidine, x 400. **Lower panel (A-G);** IV. Sequencing chromatogram of *MYOD1* (L122R, T>G or T>TG) are shown with reference sequences (Ref) and mutation showing forward (F) and reverse (R) sequencing reads. Arrow indicates nucleotide position which harbor mutation

**Supplementary Table 1:** Clinicopathological features, including results of *MYOD1* (L122R) mutations in 21 cases of spindle cell and sclerosing rhabdomyosarcomas.

**Supplementary Table 2:** *MYOD1* Mutation status, Treatment and Outcomes in 21 cases of spindle cell and sclerosing rhabdomyosarcomas.

**Supplementary Table 3:** List of various antibody markers used in the present study.

**Supplementary Table 4.** Primer details used for mutation analysis of *MYOD1* and *PIK3CA*.
