# Supplementary material for: MYOD1 (L122R) mutations are associated with spindle cell and sclerosing rhabdomyosarcomas with aggressive clinical outcomes
Source: Mod Pathol. 2016 Aug 26;29(12):1532–40. doi: 10.1038/modpathol.2016.144 (PMC5133269; doi:10.1038/modpathol.2016.144)
Supplement: Supplementary Table 1 [file modpathol2016144x2.doc]

**Supplementary Table 1.** Clinicopathologic features, including results of *MYOD1* p.L122R mutations in 21 cases of spindle cell and sclerosing rhabdomyosarcomas.

| **Sr No.** | **Age/Sex** | **Site** | **Histopathologic subtype** | **Immunohistochemical Profile** | ***MYOD1*(L122R)** |
| --- | --- | --- | --- | --- | --- |
| 1 | 28/M | H and N (Infratemporal) | Sclerosing | Desmin-P, MYOD1-P, Myogenin-P, SMA-P | Mutant (Heterozygous) |
| 2 | 26/F | H and N (Orbit) | Sclerosing | Desmin-P, MYOD1-P, Myogenin-P, SMA-P | Mutant (Homozygous) |
| *3 | 17/M | Thigh | Sclerosing | Desmin-P, MYOD1-P, Myogenin-P | Mutant (Heterozygous) |
| 4 | 26/M | H and N (Buccal mucosa, Orbit) | Sclerosing | Desmin-P, MYOD1-P | Mutant (Heterozygous) |
| 5 | 11/M | H and N (Maxilla) | Sclerosing | Desmin-P, MYOD1-P, Myogenin-P | Mutant (Heterozygous) |
| 6 | 24/M | H and N (Alveolus) | Sclerosing | Desmin-P, MYOD1-P, Myogenin-P, SMA-P | Mutant (Heterozygous) |
| 7 | 25/M | H and N (Face) | Sclerosing | Desmin-P, MYOD1-P, Myogenin-P, SMA-P | Mutant (Heterozygous) |
| 8 | 25/M | Elbow | Spindle cell | Desmin-P, MYOD1-P, Myogenin-P, SMA-N, | Mutant (Homozygous) |
| 9 | 19/M | H and N (Oral cavity, maxilla) | Spindle cell | Desmin-P, MYOD1-P Myogenin-P, SMA-N | Mutant (Heterozygous) |
| #10 | 30/M | Chest wall | Spindle cell | Desmin-P, MYOD1-P, Myogenin-P, SMA-P | Mutant (Heterozygous) |
| 11 | 7/F | Abdomen(Mesentery) | Spindle cell | Desmin-P, MYOD1-P ,Myogenin-P | Wild Type |
| 12 | 17/M | Retroperitoneum | Spindle cell | Desmin-P, MYOD1-N, Myogenin-P | Wild Type |
| 13 | 2/M | H and N (Soft palate) | Spindle cell | Desmin-P, MYOD1-P, Myogenin-P, Myoglobin-P | Wild Type |
| 14 | 66/M | Retroperitoneum | Spindle cell | Desmin-P, MYOD1-P, Myogenin-P, SMA-N | Wild Type |
| 15 | 21/F | H and N (Face) | Spindle cell | Desmin-P, Myogenin-P, SMA-N | Wild Type |
| 16 | 7/M | H and N (Parotid) | Sclerosing | Desmin-P, MYOD1-P, Myogenin-P, SMA-N | Wild Type |
| 17 | 3/M | H and N (Orbit) | Sclerosing | Desmin-P, MYOD1-P, Myogenin-P | Wild Type |
| 18 | 19/M | Paratesticular | Spindle cell | Desmin-P, MYOD1-P, Myogenin-N | Wild Type |
| 19 | 17/M | Paratesticular | Spindle cell | Desmin-P, Myogenin-P, SMA-P | Wild Type |
| ##20 | 20/M | Paratesticular | Spindle cell | Desmin-P, MYOD1-P, Myogenin-N | Wild Type |
| 21 | 2/M | Paratesticular | Spindle cell | Desmin-P, MYOD1-N, Myoglobin-P | Wild Type |

F: Female, M: Male, H and N: Head and Neck, P: Positive, N: Negative, SMA: Smooth muscle actin. Cases1, 2, 5 and 16 also displayed focal spindle cells.
