# Supplementary material for: MYOD1 (L122R) mutations are associated with spindle cell and sclerosing rhabdomyosarcomas with aggressive clinical outcomes
Source: Mod Pathol. 2016 Aug 26;29(12):1532–40. doi: 10.1038/modpathol.2016.144 (PMC5133269; doi:10.1038/modpathol.2016.144)
Supplement: Supplementary Table 2 [file modpathol2016144x3.doc]

**Supplementary Table 2.** *MYOD1* Mutation status, Treatment and Outcomes in 21 cases of spindle cell and sclerosing rhabdomyosarcomas.

| **Sr No.** | **Age/Sex** | ***MYOD1*(L122R)** | **Treatment** | **Outcome** | **Recurrences/metastasis** | **Follow-up(months)** |
| --- | --- | --- | --- | --- | --- | --- |
| 1 | 28/M | Mutant (Heterozygous) | Surgical resection +CT+RT | Alive with disease | Mets (spine skeletal) | 14 |
| 2 | 26/F | Mutant (Homozygous) | Surgical resection + RT | Alive with disease | Mets (lung) | 1 |
| *3 | 17/M | Mutant (Heterozygous) | Surgical resection,CT+RT | NK | NK | NK |
| 4 | 26/M | Mutant (Heterozygous) | Surgical resection + CT | Alive with disease | Recurrences | 23 |
| 5 | 11/M | Mutant (Heterozygous) | Surgical resection + CT | Alive with disease | Mets (lung) | 22 |
| 6 | 24/M | Mutant (Heterozygous) | Surgical resection,CT+RT | Free of disease | Recurrence | 50 |
| 7 | 25/M | Mutant (Heterozygous) | Surgical resection,CT+RT | Alive with disease | NK | 6 |
| 8 | 25/M | Mutant (Homozygous) | Surgical resection,CT+RT | Free of disease | NK | 6 |
| 9 | 19/M | Mutant (Heterozygous) | Surgical resection,CT+RT | Alive with disease | Residual disease | 12 |
| #10 | 30/M | Mutant (Heterozygous) | Surgical resection + RT | Alive with disease | Recurrence | 5 |
| 11 | 7/F | Wild Type | Surgical resection + CT | Free of disease | NK | 40 |
| 12 | 17/M | Wild Type | Surgical resection,CT+RT | Free of disease | NK | 7 |
| 13 | 2/M | Wild Type | NK | NK | NK | NK |
| 14 | 66/M | Wild Type | NK | NK | NK | NK |
| 15 | 21/F | Wild Type | Surgical resection + CT | NK | NK | NK |
| 16 | 7/M | Wild Type | Surgical resection,CT+RT | Free of disease | NK | 28 |
| 17 | 3/M | Wild Type | Surgical resection + CT | Free of disease | NK | 33 |
| 18 | 19/M | Wild Type | Surgical resection,CT+RT | Alive with disease | Mets (node, lungs, skeletal) | 33 |
| 19 | 17/M | Wild Type | Surgical resection + CT | Free of disease | Mets (Node) | 31 |
| ##20 | 20/M | Wild Type | Surgical resection + CT | Alive with disease | Mets | 12 |
| 21 | 2/M | Wild Type | Surgical resection + CT | Free of disease | NK | 24 |

RT: Radiotherapy, CT: Chemotherapy, mets: metastasis, NK: Not Known. *: Presented with pulmonary and inguinal lymph node metastasis, #Presented with pulmonary metastasis. ##: Presented with pulmonary and bone marrow metastasis. Cases 1, 5, 8, 9 and 10 constitute as cases from our previously published study.^12^ Cases1, 2, 5 and 16 also displayed focal spindle cells
