# Supplementary figures and images for: MYOD1 (L122R) mutations are associated with spindle cell and sclerosing rhabdomyosarcomas with aggressive clinical outcomes
Source: Mod Pathol. 2016 Aug 26;29(12):1532–40. doi: 10.1038/modpathol.2016.144 (PMC5133269; doi:10.1038/modpathol.2016.144)

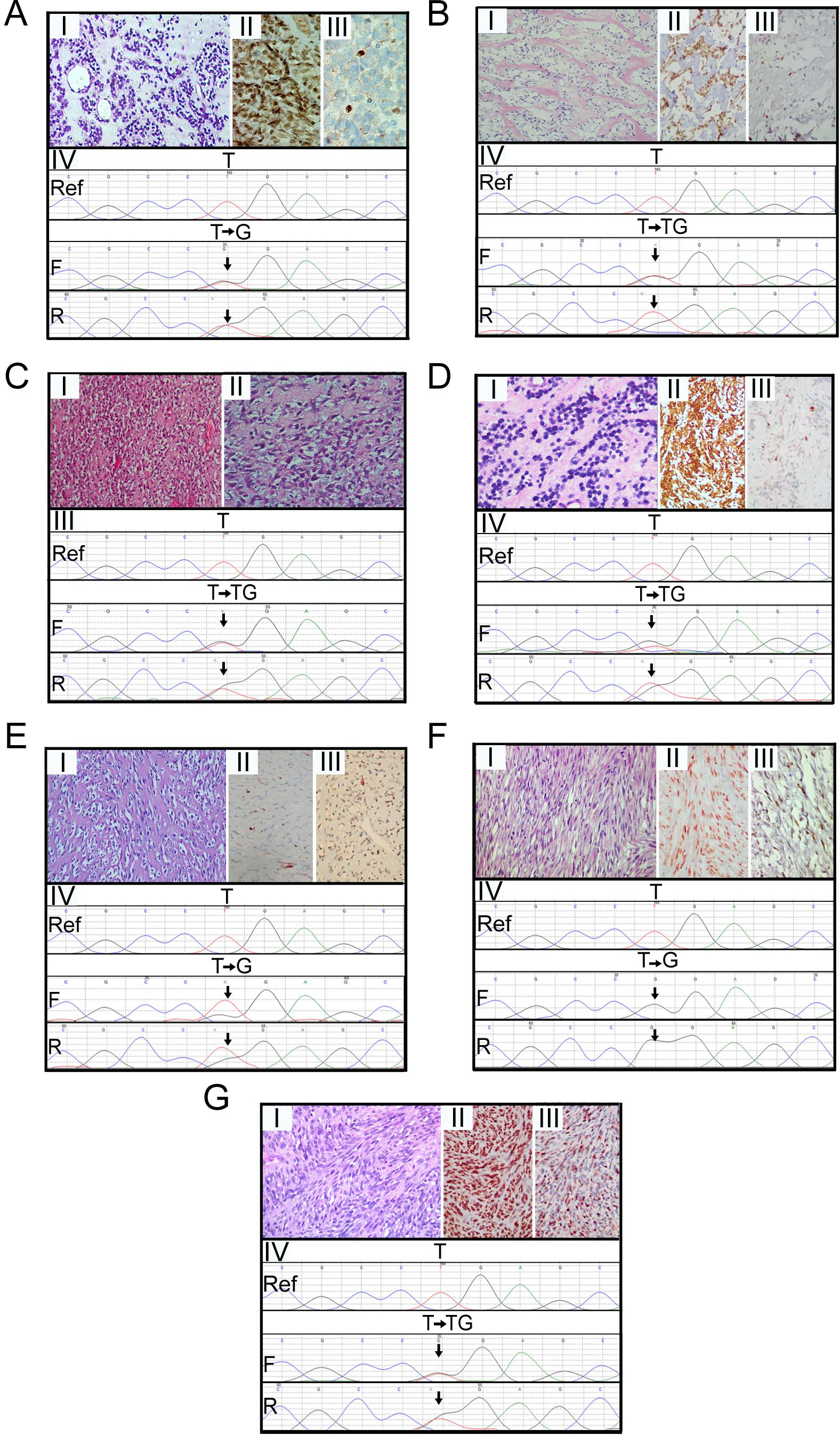

Supplement: Supplementary Table 3 [file modpathol2016144x4.tif]
