# Supplementary material for: MYOD1 (L122R) mutations are associated with spindle cell and sclerosing rhabdomyosarcomas with aggressive clinical outcomes
Source: Mod Pathol. 2016 Aug 26;29(12):1532–40. doi: 10.1038/modpathol.2016.144 (PMC5133269; doi:10.1038/modpathol.2016.144)
Supplement: Supplementary Table 4 [file modpathol2016144x5.doc]

**Supplementary Table 3.** List of various antibody markers used in the present study.

| **Sr No.** | **Antibody Marker** | **Clonality, Clone** | **Dilution** | **Antigen Retrieval** | **Manufacturer** |
| --- | --- | --- | --- | --- | --- |
| 1 | Desmin | Monoclonal, D33 | 1:200 | Heat. Pascal (Tris-EDTA) | Dako, Glostrup, Denmark |
| 2 | MYOD1 | Monoclonal, 5.8A | 1:40 | Heat. Pascal (Tris-EDTA) | Dako |
| 3 | Myogenin | Monoclonal, L026 | 1:50 | Heat. Pascal (Sodium citrate) | Leica, Novacastra, New Castle upon Tyne, England |
| 4 | Smooth muscle actin (SMA) | Monoclonal, 1A4 | 1:400 | Heat. Pascal (Tris-EDTA) | Dako |
