# Supplementary material for: MYOD1 (L122R) mutations are associated with spindle cell and sclerosing rhabdomyosarcomas with aggressive clinical outcomes
Source: Mod Pathol. 2016 Aug 26;29(12):1532–40. doi: 10.1038/modpathol.2016.144 (PMC5133269; doi:10.1038/modpathol.2016.144)
Supplement: Supplementary Figure 1 [file modpathol2016144x6.doc]

**Supplementary Table 4.** Primer details used for mutation analysis of *MYOD1* and *PIK3CA*.

| **Primer Name** | **Primer Details** | **Primer Sequence (5ˈ-----------3ˈ)** |
| --- | --- | --- |
| OAD0975 | *MyoD1* (L122) F | CAAGCGCAAGACCACCAAC |
| OAD0976 | *MyoD1* (L122) R | GGTTTGGATTGCTCGACGTG |
| OAD963 | *PIK3CA* (E542/E545) F | CAATGAATTAAGGGAAAATGA |
| OAD1227 | *PIK3CA* (E542/E545) R | AGATCAGCCAAATTCAGTTA |
| OAD1087 | *PIK3CA* (H1047) F | AGCCTTAGATAAAACTGAGCA |
| OAD1088 | *PIK3CA* (H1047) R | TCAATGCATGCTGTTTAATTG |
